# Genome sequences of three *Streptomyces* isolated from the soil of soybean field in Tsuruoka, Japan

**DOI:** 10.1128/mra.01059-24

**Published:** 2024-11-27

**Authors:** Ryuki Sato, Rena Saito, Tomoki Takeda, Nobuhiro Sasaki, Satoshi Yuzawa, Natsumi Saito, Kazuharu Arakawa

**Affiliations:** 1 Institute for Advanced Biosciences, Keio University, Tsuruoka, Yamagata, Japan; 2 Systems Biology Program, Graduate School of Media and Governance, Keio University, Fujisawa, Kanagawa, Japan; 3 National Institute of Technology (KOSEN), Tsuruoka College, Tsuruoka, Yamagata, Japan; 4 Faculty of Environment and Information Studies, Keio University, Fujisawa, Kanagawa, Japan; University of Strathclyde, Glasgow, United Kingdom

**Keywords:** *Streptomyces*, complete genome, nanopore sequencing, soybean field

## Abstract

*Streptomyces* are Gram-positive bacteria typically found in the soil, with very large genomes and high GC content, and are known to produce a wide range of secondary metabolites. We isolated and sequenced the genomes of three *Streptomyces* bacteria from the soil of soybean field in Tsuruoka, Japan.

## ANNOUNCEMENT

We have isolated three *Streptomyces* bacteria (1) from the rhizosphere soil of soybeans in a field in Tsuruoka city, Yamagata Prefecture, Japan (GPS: 38.762647, 139.811495), on 3 August 2015. The soil was crushed and dried at room temperature for at least 5 days to promote actinomycete spore formation. Actinomycetes were isolated from the dried soil by a dry-heating method. After heat treatment of the soil at 100°C for 30 min, the soil was spread on humic acid-vitamin (HV) agar medium and incubated at 27°C for 7–10 days. The composition of HV agar per liter is as follows: humic acid, 1 g; KCl, 1.71 g; Na_2_HPO_4_, 0.5 g; FeSO_4_ · 7H_2_O, 0.01 g; MgSO_4_ · 7H_2_O, 0.05 g; CaCO_3_, 0.02 g; vitamin mix (alinamin V; Alinamin Pharmaceutical Co., Ltd., Japan), 5 mL; nalidixic acid, 15 mg; cycloheximide, 50 mg; and agar, 18 g, prepared to a pH of 7.2. Actinomycetes colonies were picked and transferred onto HV agar, and purified by streaking on YS (Yeast-Starch) agar medium (per liter: yeast extract, 2 g; soluble starch, 10 g; agar, 20 g). The strains were cultured aerobically at 30°C for 7 days using YS agar medium. Following a 7-day incubation period, a single colony was transferred to 5 mL of tryptic soy broth (TSB) liquid medium supplemented with nalidixic acid and cultured for 4 days at 30 °C. Later, 1 mL of this culture was inoculated into 50 mL of TSB liquid medium with nalidixic acid and incubated for another 4 days (2). Subsequently, the cells were collected and pelletized, and the genomic DNA was extracted using NucleoBond HMW DNA Kit (Macherey-Nagel, Düren, Germany) according to the manufacturer’s protocol. Long read sequencing libraries were prepared using Rapid Barcoding Kit (SQK-RBK114.24, Oxford Nanopore Technologies) and sequenced on the GridION device using R10.4.1 Flowcell (FLO-MIN114, Oxford Nanopore Technologies). Sequenced reads were basecalled, demultiplexed, and trimmed for the adaptor sequences using Guppy 6.5.7 (super accurate mode). The quality of the long reads was assessed using Nanoplot (3), and reads longer than 10 kb (around 30–60× coverage) were adapter trimmed using Porechop version 0.2.3 (4) and were then assembled using Canu version 2.2 (5). The completeness statistics were saturated by a single round of error correction using medaka version 1.11.3 with the reads after adapter trimming. The completeness of the assembly was assessed with CheckM version 1.2.2 (6) to be 99.89% (BC10), 99.93% (BC04), and 99.86% (BC02) with automatic lineage identification (f__Streptomycetaceae [UID2048]). The linear genomes were annotated using DDBJ Fast Annotation and Submission Tool (DFAST) version 1.2.18 (7). Sequence accession numbers, sequenced reads, genome assembly, and annotation statistics are listed in Table 1. Default parameters were used except where otherwise noted.

**TABLE 1:** Genome assembly statistics and accession numbers

Bacterial species	*Streptomyces bungoensis*	*Streptomyces sennicomposti*	*Streptomyces* sp.
Isolate	E-12	C-18	E-15
Accession no.	AP038332	BAAFSZ010000001	BAAFTA010000001
		BAAFSZ010000002	BAAFTA010000002
		BAAFSZ010000003	BAAFTA010000003
BioProject ID	PRJNA1155174	PRJNA1155174	PRJNA1155174
Run ID	SRR30504527	SRR30504526	SRR30642279
Total number of reads	32,156	47,706	2,388,114
Read N50 (bp)	28,373	13,203	2,552
No. of contigs	1	3	3
Coverage	62×	37×	602×
Genome assembly size (bp)	8,270,802	8,655,445	8,652,591
Contig N50 bp (#)	8,270,802 (#1)	6,827,012 (#1)	7,170,233 (#1)
No. of CDSs (CoDing Sequence)	7,276	7,749	7,463
G + C content (%)	72.9	72.6	72.9
No. of rRNAs	18	21	18
No. of tRNAs	102	103	93

The assembled genome of isolate BC10 was identified to be *Streptomyces bungoensis* based on ANI (Average Nucleotide Identity) of 95.86% to GCA_001514215.1 (8), BC04 was identified to be *Streptomyces sennicomposti* based on ANI of 95.63% to GCA_019890635.1 (9), and that of BC02 was inconclusive at the species level (ANI of 98.35% to *Streptomyces* sp. GCF_000718775.1 (10) and no hits with ANI > 95% with defined species). ANI was calculated as part of the DFAST pipeline (7).

## Data Availability

The genome sequences reported here were deposited in DDBJ, and the raw reads were deposited in the Sequence Read Archive (SRA), under the accession numbers listed in Table 1.
